# Two-Stage System Based on a Software-Defined Radio for Stabilizing of Optical Frequency Combs in Long-Term Experiments

**DOI:** 10.3390/s140101757

**Published:** 2014-01-20

**Authors:** Martin Čížek, Václav Hucl, Jan Hrabina, Radek Šmíd, Břetislav Mikel, Josef Lazar, Ondřej Číp

**Affiliations:** Institute of Scientific Instruments of the ASCR, Královopolská 147, Brno 612 64, Czech Republic; E-Mails: treak@isibrno.cz (V.H.); shane@isibrno.cz (J.H.); smid@isibrno.cz (R.Š.); mikel@isibrno.cz (B.M.); joe@isibrno.cz (J.L.); ocip@isibrno.cz (O.Č.)

**Keywords:** optical frequency combs, digital signal processing, software-defined radio, beat note, stabilization, long-term operation

## Abstract

A passive optical resonator is a special sensor used for measurement of lengths on the nanometer and sub-nanometer scale. Astabilized optical frequency comb can provide an ultimate reference for measuring the wavelength of a tunable laser locked to the optical resonator. If we lock the repetition and offset frequencies of the comb to a high-grade radiofrequency (RF) oscillator its relative frequency stability is transferred from the RF to the optical frequency domain. Experiments in the field of precise length metrology of low-expansion materials are usually of long-term nature so it is required that the optical frequency comb stay in operation for an extended period of time. The optoelectronic closed-loop systems used for stabilization of combs are usually based on traditional analog electronic circuits processing signals from photodetectors. From an experimental point of view, these setups are very complicated and sensitive to ambient conditions, especially in the optical part, therefore maintaining long-time operation is not easy. The research presented in this paper deals with a novel approach based on digital signal processing and a software-defined radio. We describe digital signal processing algorithms intended for keeping the femtosecond optical comb in a long-time stable operation. This need arose during specialized experiments involving measurements of optical frequencies of tunable continuous-wave lasers. The resulting system is capable of keeping the comb in lock for an extensive period of time (8 days or more) with the relative stability better than 1.6 × 10^−11^.

## 1. Introduction

The work is motivated by the fact that with the advent of fully digital telecommunications in the past decade, the real-time digital signal processing in the radio frequency (RF) domain has quickly moved from theoretical outlines to engineering practice. This has allowed replacing some blocks of traditional analog electronics by software algorithms in many types of servo-loops. A similar situation is seen in the fundamental metrology of precise time and frequency. These quantities are processed very often by optical frequency combs which have become important instruments in the field of measuring optical frequencies of continuous-wave (CW) laser sources [1]. These instruments are also called optical frequency synthesizers because they are able to transfer the relative stability of RF signals to the optical frequency domain and *vice versa* [2,3].

In principle, the optical frequency comb is based on a femtosecond mode-locked laser. It generates a train of pulses, which from the signal processing point of view is an optical-frequency carrier modulated by a radio-frequency (RF) pulsed envelope. Pulse widths are typically in the range of tens of femtoseconds up to 1 ps. According to signal processing theory, such waveforms are transformed into a set of evenly spaced components in the frequency domain. The spacing of these components is equal to the repetition frequency *f_rep_* of the pulses and the offset of these pulses with respect to zero is dependent on the carrier-to-envelope frequency, the so called offset frequency *f_ceo_*. Hence the output of a system like this can be seen as a set of many lasers working synchronously at the same time at evenly spaced optical frequencies. The optical frequency of a certain spectral component *ν_i_* can be easily described by the following formula [4]:

(1) $$\nu_{i}=f_{\text{ceo}}+i\cdot f_{\text{rep}}$$

where *i* is the index of the spectral component; *f_rep_* is usually from tens to several hundreds of MHz and *f_ceo_* is in the range from 0 Hz to *f_rep_*. The width of the optical spectrum of generated femtosecond pulses depends mainly on the width of a gain spectrum profile of the active media of the mode-locked laser. Incase of an infrared Er^3+^ doped fiber femtosecond laser the output spectrum is typically 200 nm wide and finds itself around a 1550 nm central wavelength [5,6].

The transfer of the relative stability of a radiofrequency signal to the optical domain is based on this technology of femtosecond optical synthesizers [7,8]. If both repetition and offset frequencies are stable then the optical frequencies of comb spectral components *ν_i_* are stable. In case of the repetition frequency, its absolute stability value is multiplied by the order *i* of the appropriated component *ν_i_*. The absolute stability of the offset frequency has an additive contribution to the resulting absolute stability of the optical frequency *ν_i_* of this comb spectral component. As is clearly visible, the stability of the repetition frequency is more important than the offset one, but for some critical applications (*i.e.*, ion clock comparison) both frequencies should be stable as much as possible [9,10].

The femtosecond lasers with passive mode-lock are mainly used in the field of metrology of precise frequency and time. On basis of the theoretical expression (1) it seems to be quite easy to generate a certain optical frequency *ν_i_*, but from the experimental point of view, these systems are very complicated, especially in the optical part, and keeping these lasers in the long-term working operation is not easy. In the case of systems built around bulk optics (*i.e.*, Ti:Sa working at 810 nm), already some small acoustic ripples or short spikes can immediately disturb the pulsed regime. Again the above-mentioned fiber-based lasers need a temperature stabilized room for long-time mode-locked operation without drops if such operation is needed.

The stability of the offset and repetition frequency is conventionally ensured by a set of two independent phase locked loop (PLL) controllers. Each of them is able to keep the phase of the appropriate signal with any radiofrequency standard source (*i.e.*, H-maser, Rb or Cs clocks, GPS disciplined oscillators) [11,12]. The relative stability of those standards is then transferred to the stability of the repetition and offset frequency of the comb. If the behavior of the femtosecond laser doesn't have dropouts then such a solution works well. In the case of long-term experiments dropouts should be expected and therefore the mentioned controllers for offset and repetition frequency must solve these exceptions. This leads to sophisticated servo-loop algorithms but commercially available controllers based on analog techniques don't have the possibility to prevent these problems [13,14]. The digital lock-ins and different controllers on the market are able to work with a high dynamic range of controlled phase but spikes or long value wander lead to dropouts of the PLL lock [14,15]. Also an important point is that these controllers are usually constructed as single-purpose devices. Those lack almost any remote controlling capabilities which make them hard to use in long-term running experiments. Another problem is these systems for controlling *f_ceo_* sometimes have only a fixed frequency setpoint like *f_rep_*/4, *etc.* [14].

Previously we experimented with using software-defined radios in realizing phase-locked loops for stabilization of combs and CW lasers [16]. If left alone these digital systems are sensitive to unwanted interference in a similar way as the above-mentioned analog controllers. We have developed new supervisory software blocks that combined with new digital signal processing algorithms, which are explained in the work, are able to keep the femtosecond optical combs in the long-term stable operation. This need arose during specialized experiments for the measurement of optical frequencies of a tunable CW laser. In our case we applied the novel digital signal processing technique in the direct and real-time detection of the refractive index of air. This example covers the CW laser which is locked to an optical cavity made from low expansion Zerodur material [17] and the optical frequency of a CW laser is monitored by the femtosecond comb. A period of 8 days of comb operation without any dropouts was needed. Therefore this comb was stabilized using our novel digital signal processing-based techniques.

## 2. Stabilization of Repetition and Offset Frequencies in Femtosecond Combs

The repetition frequency of the femtosecond laser can be easily measured by a fast photodetector at the output of the femtosecond laser. The offset frequency can be measured using the f-2f technique [18–20] which involves mixing the original output of the femtosecond laser with its 2nd harmonics and detecting the mixing product with a fast photodetector. This signal is called the f-2f signal in the following text. The process of mixing the comb spectrum with its 2nd harmonics can be mathematically described as follows:

(2) $$\begin{array}{c} 2\nu_{i}-\nu_{2i}=2(f_{\text{ceo}}+i\cdot f_{\text{rep}})-(f_{\text{ceo}}+2i\cdot f_{\text{rep}})=\\ \;=2f_{\text{ceo}}-f_{\text{ceo}}+2i\cdot f_{\text{rep}}-2i\cdot f_{\text{rep}}=\underset{͇}{f_{\text{ceo}}} \end{array}$$

In order to use the comb spectrum as a reference for optical frequency measurements it is necessary to stabilize *f_rep_* and *f_ceo_* with respect to a time or frequency standard. One possible way, which relates to the topic of this article, is by locking both the repetition and the offset frequencies of the comb to the output of a RF standard and in fact transferring its relative stability to the optical frequency domain. This standard can be for example a GPS-disciplined oscillator, a stable frequency generator based on H-maser or an atomic clock. Such relative stability transfers are usually achieved using phase or frequency-locked loops based on common analog RF electronic parts and subsystems like frequency filters, oscillators, balanced mixers, demodulators and operational amplifiers.

## 3. Methods for Digital Processing of RF Signals

In the following sections we will concentrate on implementing RF digital signal processing for building a system for long-term stabilization of optical frequency combs.

### 3.1. Phase-Locked Loop Based on Software-Defined Radio

**Figure 1.**
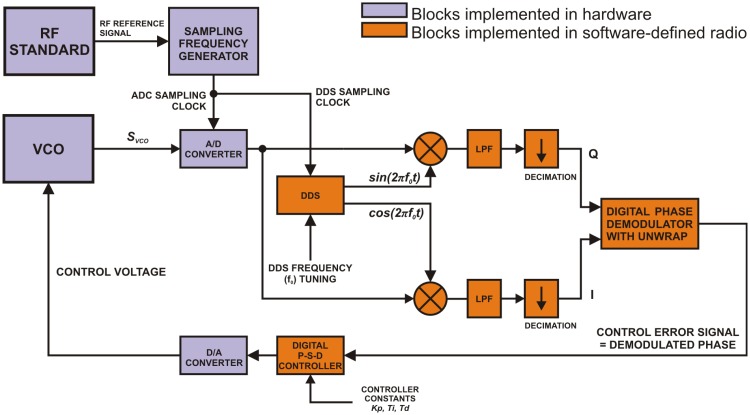
The block schematics of the software-defined radio system for digital phase locking: VCO is a voltage controlled oscillator as the example of the controlled system; LPF is the digital low pass filter; A/D and D/A converters are analog-to-digital and digital-to-analog converters, respectively; RF standard is a high-grade harmonic oscillator with ultimate stability of generated frequency; DDS is the direct-digital synthesizer.

A direct-sampling software-defined radio (SDR) algorithm described in Figure 1 was implemented on a special PC computer system in order to form a digital phase-locked loop. In the example a voltage controlled oscillator (VCO) demonstrates the system generating a harmonic signal *s_vco_* with frequency *f_vco_* that should be stabilized by this algorithm.

Samples of the input RF signal *s_vco_* digitized by a high-speed A/D converter are processed in the following way: the first step is digital filtering of the *s_vco_* signal with digital down-conversion followed by low-pass filtering (LPF). A direct digital synthesis (DDS) block generates sine and cosine waveforms used for digital down-conversion. Frequencies of these signals are equal to *f*_0_, which is the frequency setpoint of the PLL system. The in-phase signal *I* is produced by multiplying the samples of the input signal *s_vco_* with cosine waveform generated by the DDS. The quadrature *Q* signal is obtained by multiplying the same input samples with the sine waveform. According to the basics of digital signal processing theory these *I* and *Q* signals are in fact the Cartesian coordinates of a vector whose argument is equal to the instantaneous phase difference between the input harmonic signal and the DDS cosine signal. Low-pass filtering of *I* and *Q* signals is necessary in order to eliminate higher-order mixing products. In an ideal case the cut-off frequency *f_c_* of these low-pass filters should be equal to the maximum expected absolute value of the deviation of the *f_vco_* frequency from *f_0_*. The resulting *I* and *Q* signals have limited bandwidths in comparison to the original input signal *s_vco_*. This can be taken advantage of in reducing the computational overhead in the digital phase demodulator block. In order to eliminate the unnecessary data throughput, the filtered signals are decimated to a lower sampling frequency *f_sIF_* with the ultimate limit expressed by *f_sIF_* = 2*f_c_* according to the Nyquist-Shannon theorem.

Speaking about a harmonic signal we can distinguish between two representations of its instantaneous phase. If we constrain the phase to its principal value we get a discontinuous function with values in [−π, π) interval. This is the wrapped instantaneous phase of the harmonic signal. Without this constraining we get the unwrapped instantaneous phase represented as a continuous function with an infinite interval of possible values.

In our system a special form of digital phase detector that performs phase unwrapping is used. Instead of computing the phase straightforwardly from values of *I* and *Q* samples using the *arctan* function, which results in wrapped phase information, the algorithm computes the unwrapped phase value that can go beyond the [−π, π] interval. This technique is one of the advantages introduced by software-defined radios. The ability to work with the unwrapped phase theoretically extends the capture range of the realized digital phase-locked loop to a frequency interval given by the *I* and *Q* low-pass filters in the following way:

(3) $$B_{\text{CAPTURE}}=[f_{0}-f_{c};\;f_{0}+f_{c})$$

where *B_CAPTURE_* is the capture frequency interval of the PLL, *f*_0_ is the frequency generated by the DDS and *f_c_* is the cut-off frequency of the two identical low-pas filters processing the *I* and *Q* signals.

The main idea of digital phase demodulation with unwrapping is that the system remembers the initial phase of the signal. With every new sample of the processed signal this value is updated by adding it to the instantaneous difference between the actual and previously computed phase. The result of this operation is obviously an unconstrained phase value that is the unwrapped phase. Provided that the maximum absolute deviation of *f_vco_* from *f*_0_ is two times lower than the sampling frequency *f_sIF_* of the *I* and *Q* signals the phase-detecting algorithm will not miss any phase change. The process of the digital phase demodulation with unwrapping can be described using complex-number algebra. Samples of the *I* and *Q* signals are in fact real and imaginary parts of a complex signal *x*:

(4) $$\begin{array}{cc} x(n)=I(n)+jQ(n) & n=0,1,2\ldots N \end{array}$$

where *I*(*n*) is the *n*-th sample of the *I* signal, *Q*(*n*) is the *n*-th sample of the *Q* signal and *j* is the imaginary unit. The phase difference between consecutive samples ΔΦ can be computed as the argument of the scalar product of the current *x* sample and the complex conjugate of the previous *x* sample:

(5) $$\begin{array}{cc} \Delta \Phi (n)=\arg(x(n)\cdot x^{*}(n-1)) & n=1,2,3\ldots N \end{array}$$

Putting this all together the phase detection with unwrapping can be mathematically expressed as follows:

(6) $$\begin{array}{l} \Phi (0)=\arg(x(0))\\ \Phi (n)=\overset{n}{\underset{m-1}{\text{∑}}}\arg(x(m)\cdot x^{*}(m-1))+\Phi (0)\;n=1,2,3\ldots N \end{array}$$

where Φ(0) is the initial phase and Φ(*n*) is the *n*-th unwrapped phase sample and *x*(*m*) is the *m*-th sample of the complex signal, which was defined earlier in Equation (4).

In the next stage the detected phase Φ(*n*) is used as the input error signal for a proportional-summation-difference (P-S-D) controller, which is in fact a discrete-time (digital) equivalent of an analog proportional-integral-derivative (P-I-D) controller. The output of the P-S-D controller is then converted back to the analog form using a D/A converter.

**Figure 2.**
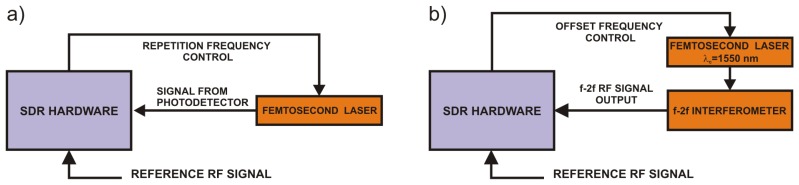
The block schematics of the setup for locking (**a**) the repetition frequency; (**b**) the offset frequency of the comb to a precise RF standard.

If we use this system in a closed loop with a general voltage-controlled oscillator we get a digital phase-locked loop, where the reference signal is given by the output of the DDS block. If all sampling clocks in the digital system are derived from a precise RF standard, the relative stability of the frequency standard will be transferred to the relative frequency stability of the VCO. The structure described in Figure 1 can be used as a versatile digital phase-locked loop controller. Instead of a general VCO we can control the repetition or offset frequency of an optical frequency comb as pictured in Figure 2.

In real-world digital implementations of the above-described techniques one may encounter issues connected with accumulating numerical rounding, detection and phase noise. On the other hand, for sufficient numbers of integrated samples, and numeric integration is what actually happens in Equation (6), these types of noise are usually considered as white ones with the mean value equal to zero [21,22]. Moreover if this digital demodulator is used in a closed-loop control application together with an integrator controller the feedback loop will cancel the effects of unwanted DC offsets accumulated due to numeric and detection noises.

### 3.2. Frequency-Locked Loop Based on FFT

Prior to starting the loop operation, for setups described in Figures 1 and 2 it is necessary to steer the controlled frequency into the capture range of the PLL. Additional frequency-locked loops (FLL) are useful for this task as well as for long-term supervising of stabilized optical frequency combs.

**Figure 3.**
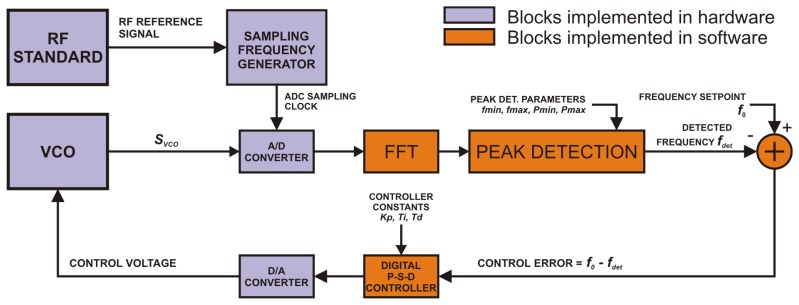
The block schematics of the frequency-locked loop based on FFT.

As shown in Figure 3 we implemented a software algorithm for frequency-locked loops based on continuous analysis of the input signal spectrum computed by the fast Fourier transform (FFT) algorithm.

Frequency domain samples computed by FFT are processed by a peak-detecting algorithm. Its input parameters are the minimum and maximum frequency *f_min_* and *f_max_* together with the minimum and maximum expected power *P_min_* and *P_max_* of the detected peak. The algorithm then simply searches for the maximum spectral peak within boundaries given by the above parameters. This allows discriminating interferences in noisy signals like for example f-2f used in offset frequency detection. The frequency resolution *Δf* of the digital FLL is given by the order *N* of the FFT and the input sampling frequency *f_s_* by the following equation:

(7) $$\Delta f=\frac{f_{s}}{N}$$

The output sampling frequency is limited mainly by the computational complexity of the FFT which generally grows with the chosen order *N*. As the evaluation of the FFT computational complexity is not in the scope of this paper one can find more information on this topic in [23].

### 3.3. System for Long-Term Stabilization of Optical Frequency Comb Based on FLL and PLL

**Figure 4.**
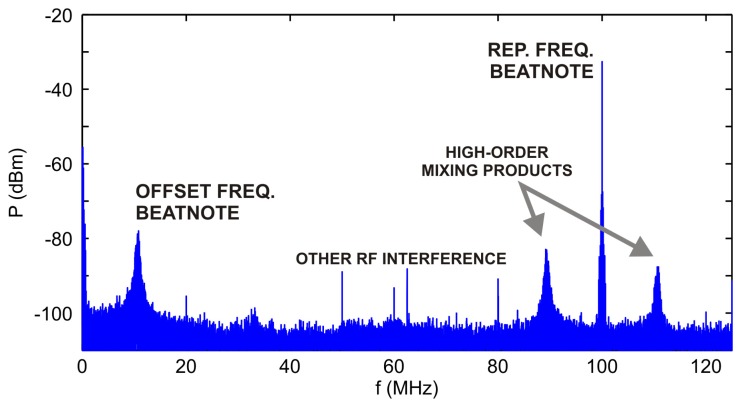
A typical power spectrum of the f-2f signal produced by an f-2f stage connected to an optical frequency comb working with 100 MHz repetition frequency.

Experiments in the field metrology of lengths described in [17,24–26] usually require uninterrupted operation of the optical frequency comb for an extended period of time. Besides the stabilization itself, the system has to deal with various interferences that are likely to occur. Since the RF signal generated by the photodetector monitoring the repetition frequency of the femtosecond laser usually has a high SNR there are not many problems with long-term stabilization. Second (or higher) order controller works good enough to eliminate temperature drifts *etc.* A more complicated situation arises if we stabilize the offset frequency by processing the RF output of the f-2f interferometer as pictured in Figure 4. Not only temperature drifts affect the SNR of the useful beat-note signal but also unwanted higher order mixing or cross-modulation products may appear or vanish over time.

By integrating digital frequency-locked loop and a digital phase-locked loop into a single system we get a setup useful for long-term stabilization of an optical frequency comb. The frequency-locking part of the system, which was described in the previous chapter, acts as a supervisor for coarse stabilization in cases when PLL is not working. The phase-locked loop is used for fine phase stabilization.

**Figure 5.**
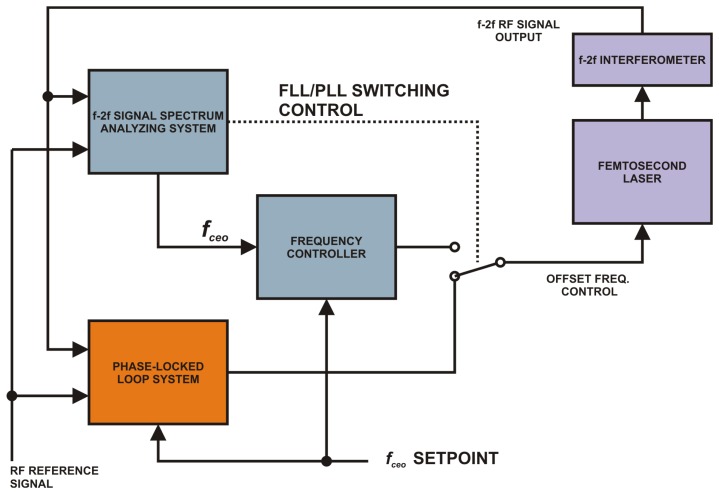
The block schematics of the system for a long-term stabilization of the offset frequency of the optical frequency comb.

The operation of the setup described in Figure 5 has three possible scenarios:

(1) **System initialization.** The PLL block is not working. The FLL part of the system is active. The user enters the desired frequency setpoint. The frequency analyzing block continuously looks for the spectral peak of the beat note in the f-2f signal and outputs the measured offset frequency. This value is used for computing the error signal in the frequency controller that steers the offset frequency into the capture range of the PLL. Once the capture range is reached the control of the offset frequency is switched to the PLL.
(2) **PLL is locked.** FLL is inactive. The software spectral analyzer (a part of the FLL loop) is just monitoring the frequency and the amplitude of the beat note. If the amplitude goes below a certain threshold the control is switched back to the frequency-locked loop and an error is reported to the user.
(3) **PLL goes out of lock.** Due to some serious interference the frequency of the beat note moved outside the PLL lock range. The PLL is switched off, an error is reported, the frequency controller steers the frequency back into the PLL capture range and turns the PLL back on.

## 4. Experimental Implementation and Results

A pilot experiment was conducted to stabilize the offset frequency of the optical frequency comb with respect to a precise RF reference which was a 10 MHz GPS-disciplined oscillator with a better than 10^−13^ long-term stability. We used an optical frequency comb with *f_rep_* = 100 MHz and central wavelength λ*_c_* = 1550 nm. During the whole long-term measurement described below, *f_rep_* of the comb was stabilized using a commercial PLL controller with 10^−11^ relative short-term stability (1 s averaging time). The comb was equipped with an f-2f interferometer for measuring of *f_ceo_*.

The digital signal processing was implemented in special computer software running on two PCs based on 8-core CPUs. The time critical parts of the software were programmed in C++ language. For FFT calculations highly optimized public-domain FFTW libraries were used. The user interface was developed in the LabView™ (National Instruments Corporation, Austin, TX, USA) environment.

The first computer used for continuous spectral analysis and frequency locking was equipped with a fast 12-bit A/D card able to work with sampling frequencies up to 250 MHz. A 250,000-point FFT algorithm was used which resulted in 1 kHz resolution in the frequency range from 0 to 125 MHz and the total computational overhead allowed maximum 40 Hz output sampling frequency.

The software-defined radio algorithm for phase detection and locking was implemented on a second computer equipped with a similar 12-bit A/D card and a 14-bit D/A card. The resolution of the digital phase detector depends on the resolution of the A/D converter and also on the extent of utilization of its input dynamic range. If the detector processes a full-scale signal quantized with 12-bit resolution the mean phase resolution is approximately 3.836 × 10^−4^ rad. For signals utilizing only a part of the A/D converter dynamic range the situation is worse. Assuming that for small angles *atan* is nearly a linear function we can state that if the input signal covers only a 1/*k* part of the dynamic range the resolution of the digital phase detector for such signal will be *k*-times worse than that of the detector processing a full-scale input signal. For this reason additional analog matching using RF amplifiers and band-pass filters at the A/D converter input is required.

**Figure 6.**
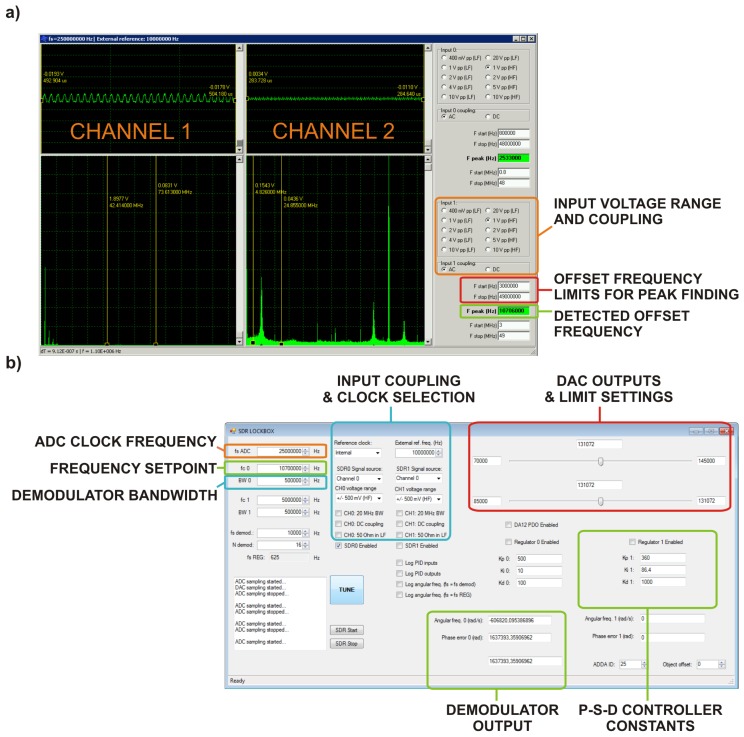
Labeled screenshots of graphical user interfaces (GUIs) of developed software applications for implementing a combined FLL and PLL system: (**a**) software for FLL control; (**b**) software for PLL control. Since the A/D converter boards have 2 input channels all the controls in the in the GUIs are also doubled.

The maximum output sampling frequency of the samples demodulated by the SDR was approx. 40 kHz with 40% of computing overhead at the given hardware configuration. The communication between the computers took place using TCP/IP protocol over a dedicated branch of Ethernet network. Implemented software applications for frequency locking using FFT and for phase locking using SDR are shown in Figure 6.

The fact that the sampling frequencies of the A/D and D/A converters were derived from the external reference ensured that all signal processing was done in the time base directly bound to the precise RF standard. This resulted in transferring its RF domain relative stability to the optical frequency domain relative stability of the comb in the 1550 nm band.

Frequency stability of *f_ceo_* was investigated using a RF frequency counter, which had its time base also locked to the RF standard. Allan deviations of *f_ceo_* were then computed for integration times up to 3.5 × 10^5^ s from data measured during eight days with 1 s gating period of the counter.

**Figure 7.**
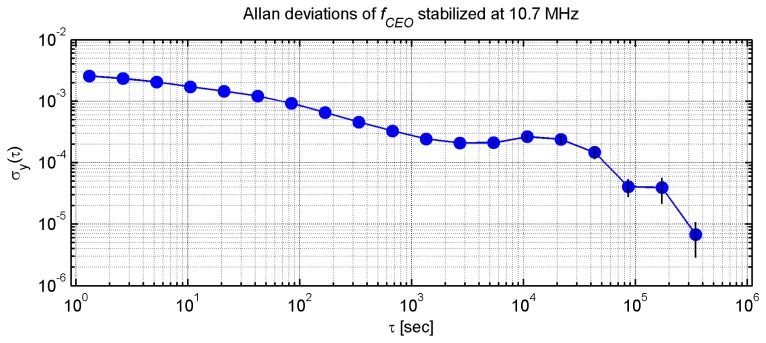
Allan deviations of the offset frequency of the comb stabilized at 10.7 MHz by software-defined radio based PLL and FLL measured for approximately eight days.

Figure 7 shows the Allan deviations computed for *f_ceo_* stabilized at 10.7 MHz. The lowest short-term Allan deviation of *f_ceo_* is 2 × 10^−4^ for 2,200 s integration time. This value is given mainly by the short-term performance of the PLL stabilization. Taking into account Equation (1) describing the comb spectrum we can evaluate how the stability of frequencies in RF domain transforms to the stability of the comb in the optical domain. Since *f_ceo_* is an absolute offset of the comb spectrum from zero, we can assume that the 2 × 10^−4^ measured relative stability this frequency transforms to 1 × 10^−11^ short-term relative stability of the optical frequency comb provided its central wavelength is 1550 nm. Next to this short-term valley a local maximum of 3.2 × 10^−4^ can be seen around approx. 1 × 10^4^ s. This relative stability value 3.2 × 10^−4^ in RF transforms to 1.6 × 10^−11^ in the optical domain. The integrating time value of the local maximum corresponds with the actual mean time between the occurrences of relocking of the system by the FLL described in the previous chapter. A long-term relative stability minimum of 7 × 10^−6^ can be seen at 3.5 × 10^5^ s. Again, this long-term relative stability of the RF signal frequency can be transformed to the relative stability of the comb working in the optical domain. In this case 7 × 10^−6^ transforms to 3.5 × 10^−13^ relative stability in the 1,550 nm band.

Besides the instabilities given by the physical nature of the optical frequency comb, phase jitters introduced by the time base of the A/D and D/A converters and phase jitters of the counter time base itself also have to be considered. For this reason we also evaluated relative deviations between the frequency of a reference test signal measured by the counter and the same frequency measured by special software utilizing the A/D converter card. Observed short-term relative deviations (1 s averaging time) were on the order of 10^−11^.

Taking into account capabilities of the available RF laboratory equipment and all the measured instabilities we can state that using our stabilization system we are able to achieve 1.6 × 10^−11^ or better relative stability of the comb for times longer than 10^3^ s.

## 5. Conclusions

The presented setup was used as a proof of concept for a system for long-term stabilization of an optical frequency comb. The technology of digital stabilization can be seen as very promising because it simplifies the design of the RF equipment. The signal processing blocks implemented in software simply cannot introduce unwanted phenomena like thermal noise, voltage drifts *etc.* Obtained results show that using combined digital FLL and PLL we are able to stabilize the optical frequency to better than 1.6 × 10^−11^ order in long-term running experiments lasting for eight days or more. It was also shown that a high-end personal computer equipped with necessary high-speed analog-to-digital and digital-to-analog converter boards can be used for real-time digital RF signal processing and run SDR applications.

However, the above-mentioned 100% computer-based approach is limited in speed. We must consider computation delays if we want to utilize computers in closed control loops. Our experimental computer systems exhibited approx. 20 ms constant delay between the A/D converter input and the D/A converter output, which was caused mainly by the software-defined radio processing and data buffering. Only relatively slow-response control loops with bandwidths up to 50 Hz can be realized. This behavior is not a big problem for the supervisory frequency locking system, which can be relatively slow. To push the bandwidth of the PLL beyond this low-frequency limit a dedicated telecommunication integrated circuit (IC) or a FPGA should be used for implementing the software-defined radio. Systems based on specialized ICs or FPGAs can reach MHz order of magnitude bandwidths.
